# Nanometer-Scale Sizing Accuracy of Particle Suspensions on an Unmodified Cell Phone Using Elastic Light Scattering

**DOI:** 10.1371/journal.pone.0046030

**Published:** 2012-10-02

**Authors:** Zachary J. Smith, Kaiqin Chu, Sebastian Wachsmann-Hogiu

**Affiliations:** 1 Center for Biophotonics Science and Technology, University of California Davis, Sacramento, California, United States of America; 2 Department of Pathology and Laboratory Medicine, University of California Davis, Sacramento, California, United States of America; University of Zurich, Switzerland

## Abstract

We report on the construction of a Fourier plane imaging system attached to a cell phone. By illuminating particle suspensions with a collimated beam from an inexpensive diode laser, angularly resolved scattering patterns are imaged by the phone's camera. Analyzing these patterns with Mie theory results in predictions of size distributions of the particles in suspension. Despite using consumer grade electronics, we extracted size distributions of sphere suspensions with better than 20 nm accuracy in determining the mean size. We also show results from milk, yeast, and blood cells. Performing these measurements on a portable device presents opportunities for field-testing of food quality, process monitoring, and medical diagnosis.

## Introduction

Mobile technologies have been advancing at a rapid pace, with current mobile platforms' computing power approaching that of desktop machines. These advances in device computing have come alongside progress in mobile imaging technology, with current cell phone cameras using sophisticated back-thinned CMOS sensors coupled to high quality optics with relatively high numerical apertures. This progress has led several groups to explore the possibility of performing medical diagnostics, such as microscopic imaging [1]–[3], cell counting [4], and spectroscopy [2], using mobile devices. We present in this paper an attachment to a cellular phone that allows for accurate sizing of particles using elastic light scattering.

Elastic light scattering has been used for several decades as an important research tool in physical sciences, medicine, and process control. In particular, light scattering has seen significant interest as a noninvasive optical tool to characterize morphological parameters of biological samples, including morphology of red blood cells [5], alterations in mitochondrial morphologies [6], [7], detection of cancers [8] and precancerous changes [9], as well as nanoscale alterations due to the field effect of cancer [10]. Advantages of scatter-based sizing over image-based sizing are ease of use and sizing accuracies well below the diffraction limit for imaging. Both of these advantages make the development of a portable particle sizer an attractive proposition.

## Results and Discussion

### Polystyrene Beads

**Figure 1:**
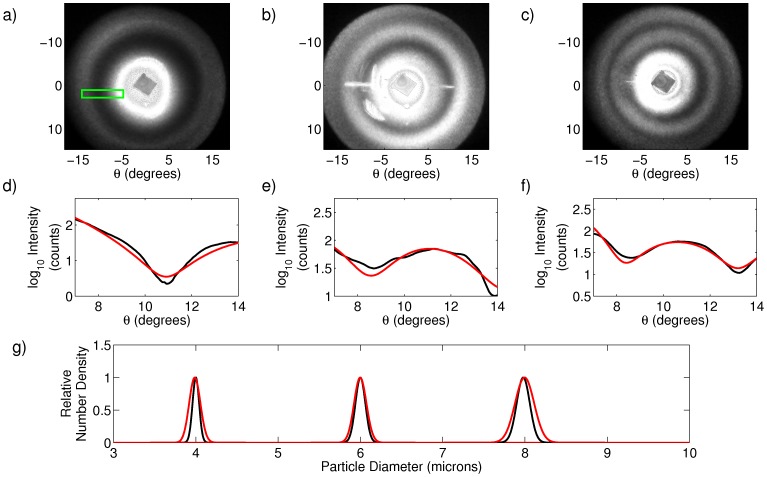
Scattering analysis of polystyrene sphere suspensions. (a)–(c) Raw scattering data from 4, 6, and 8 micron particle suspensions, respectively. The green box in (a) shows the size and shape of the area within each image from which curves in (d)–(f) were calculated. (d)–(f) One dimensional cut throughs of scattering data from 4, 6, and 8 micron particle suspensions, respectively. Black curves are experimental data, and red curves are best fits to theory. (g) Expected (black) and predicted (red) particle size distributions (D in the text) as determined from scattering data.

Construction of the cell-phone based light scattering device is detailed in the Materials and Methods section. To validate the performance of the system, we measured suspensions of NIST-traceable polystyrene beads of known size (Duke Standards 4000 series, ThermoFisher Scientific, Waltham, MA) with nominal mean diameters of 4, 6, and 8 microns. Representative scattering images are shown in Figure 1 (a)–(c), where the angle 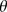 in the figures refers to the scattering angle. In Figure 1 (d)–(f) we show one dimensional cut-throughs of the experimental data in black, along with the best fit to the data in red, using the data fitting technique described in the Materials and Methods section. The green rectangle in Figure 1 (a) shows a representative area from which the cut-throughs were taken. The area is slightly offset to avoid the strong background due to the cuvette that is present in the image. In Figure 1 (g) we show the recovered particle size distributions from the experimental data for each of the three bead suspensions in red. Alongside these predictions we plot the expected size distribution as provided by the bead manufacturer in black. We repeated the size prediction 10 times, using different regions within an image and using multiple images, with the average particle size distribution predictions shown in Table 1. In all cases the predicted mean diameters agree with the expected means to within 16 nm, with an average error across the three populations of 8 nm. The precision of the measurements of the means ranged from 4 to 8 nm, showcasing the quality of the data and stability of the fits. We underscore here that the nanometer scale accuracy of optical scattering measurements is one of the signal benefits of the technique compared to optical microscopy. We note that we predict the distribution means more accurately than their widths. The width of the distribution mainly influences the depth of modulation of the fringes rather than their positions. The use of an 8 bit image sensor means that we have a difficult time distinguishing subtle variations in the modulation depth and limits both our accuracy and precision in determining this parameter.

**Table 1:** Distribution fit parameters for polystyrene bead data extracted from experimental data versus values provided by the manufacturer.

Mfg.'s Specifications		Predicted Values	
1–2 4–5 *μ*	*σ*	*μ*	*σ*
4.000	0.04	3.984 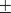 0.008	0.054 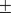 0.050
6.000	0.06	5.994 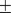 0.004	0.037 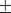 0.032
7.979	0.09	7.978 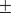 0.006	0.110 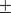 0.028

All values are given in microns.

### Dilute Suspensions of Milk in Water

**Figure 2:**
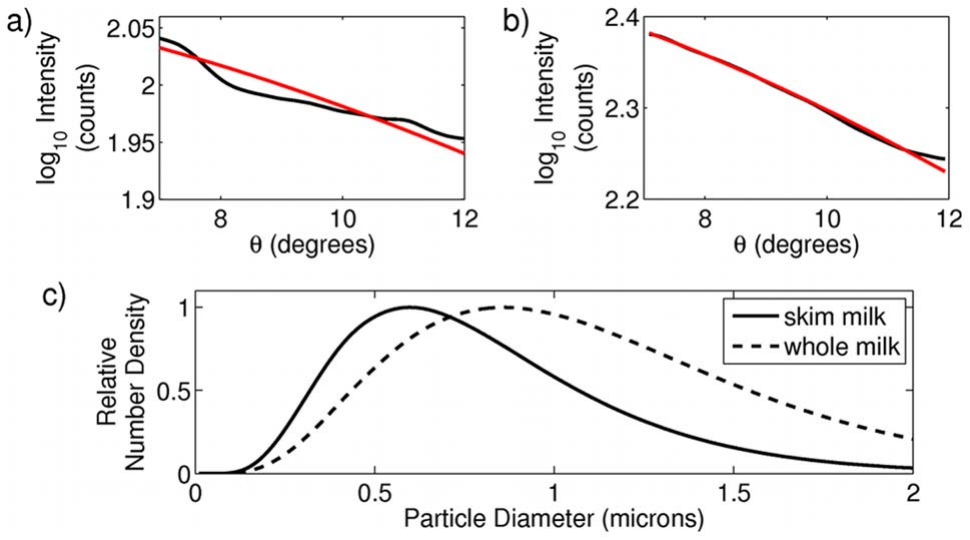
Scattering analysis of skim and whole fat milk. (a) and (b) One dimensional cut throughs of scattering data from skim and whole milk, respectively. Black curves are experimental data, and red curves are best fits to theory. (c) predicted particle size distributions as determined from scattering data for skim (solid line) and whole milk (dashed line).

We next took data from milk dissolved in water. The size and number of fat droplets in milk contribute to taste and the stability of the emulsion [11]. Protein in milk is primarily bound into small casein micelles of ranging from 50–300 nm in diameter [12], while fat droplets in homogenized milk range from 150 nm to 3 microns in diameter [11]. Skim and whole fat milk contain different proportions of protein to fat. Additionally, whole milk contains slightly larger fat droplets than are found in skim milk. We measured light scattering from a 1∶300 dilution of skim milk to water, and a 1∶40,000 dilution of whole milk to water. Assuming a log-normal distribution of particle sizes [11] and a matrix (I) of theoretical Mie intensities with radii spanning from.001 to 3 microns, we predicted particle size distributions using the method discussed above. Results from one measurement are shown in Figure 2, and an aggregate result from 10 independent predictions are summarized in Table 2. The results agree with prior reports in the literature [13], with particle size distributions dominated by the more highly scattering fat particles, and with the skim milk distribution shifted slightly to smaller sizes, indicating the different relative proportion of small protein micelles to fat globules, and an increased number of large-sized fat droplets in whole milk. Fitting with 2 distributions did not improve the quality of the fits, which we attribute to the fact that even in skim milk the distribution of casein particles contributes a negligible amount of the total scattered intensity compared to the much larger fat particles. We also see from Table 2 that the stability of the fits are significantly degraded compared to the polystyrene bead data, above. In the case of small scatterers, the scattering patterns are distinguished primarily by a slope and intercept, and as such the fits can become highly degenerate and sensitive to detector noise. Increasing the number of angles collected by our system would help to remove this degeneracy and improve fits at smaller particle sizes.

**Table 2:** Distribution fit parameters for milk, yeast, and blood cell data extracted from experimental data.

	Predicted Values	
2–3 Sample	*μ*	*σ*
skim milk	0.627 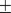 0.262	0.229 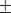 0.061
whole milk	0.835 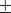 0.175	0.222 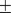 0.026
yeast	4.805 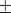 0.040	0.56 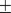 0.125
RBC (scattering)	5.634 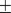 0.020	0.538 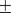 0.090
RBC (image)	5.839 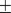 0.317	0.423 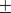 0.130

All values are given in microns.

### Suspensions of Baker’s Yeast in Water

**Figure 3:**
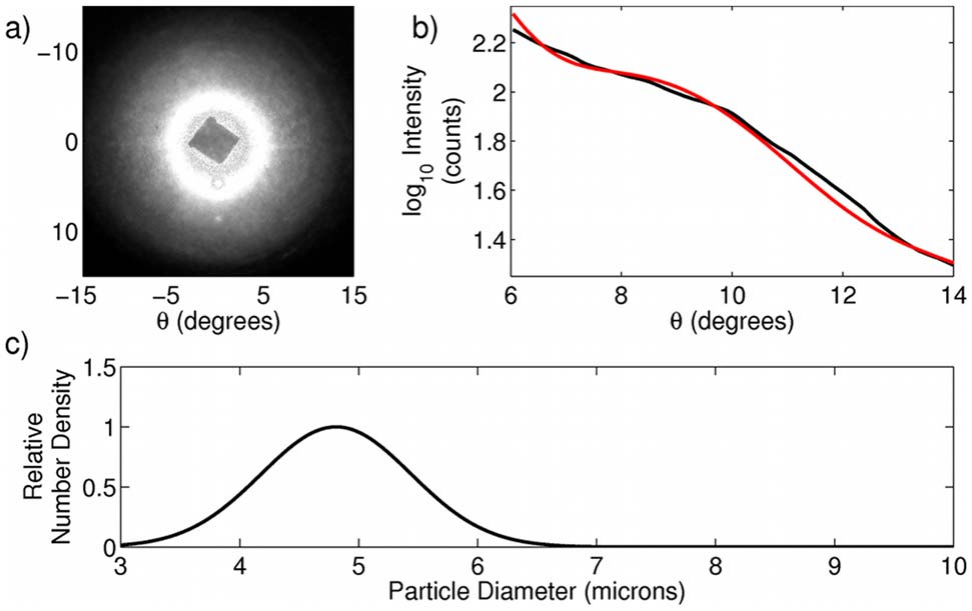
Scattering analysis of a suspension of yeast cells. (a) Raw data. (b) One dimensional cut throughs of scattering data. Black curve is experimental data, and red curve is best fit to theory. (c) predicted particle size distribution as determined from scattering data.

We also measured suspensions of baker's yeast in water. Suspensions were prepared by taking commercial baker's yeast obtained from a local supermarket (Fleischmann's Rapid Rise) and dispersing 2–3 granules in 10 mL of water and sonicating the mixture to break apart clumps of the cells. Despite the characteristic prolate spheroidal shape of baker's yeast, several experiments have confirmed that in the limit of small angle scattering, prolate spheroids, and yeast in particular, scatter as spheres with equivalent surface area [14], [15]. Yeast cell size can be a good indication of reproductive activity and overall cell health, two parameters that may be of interest to commercial or home users of yeast cultures. Using our instrument we were able to obtain a distribution of particle sizes for yeast cells (a representative fit is shown in Figure 3) that very closely agrees with previously reported distributions of yeast cell size in wild type yeast [16], with results from 10 size predictions from independent data shown in Table 2.

**Figure 4:**
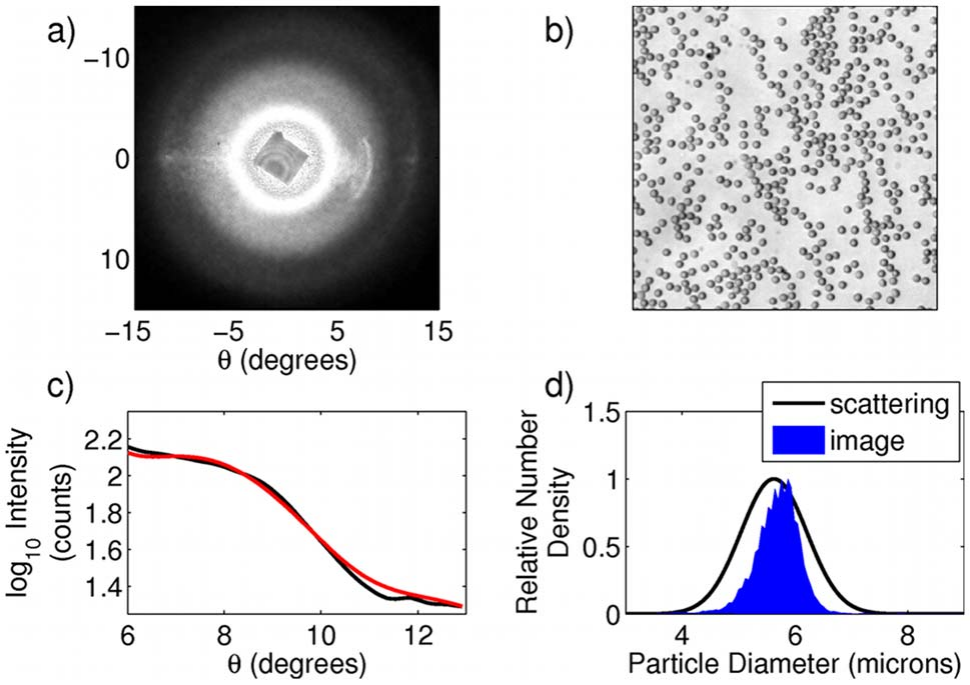
Scattering analysis of sphered red blood cells. (a) Raw scattering data. (b) Portion of a 10× microscope image of the sphered RBCs. (c) One dimensional cut throughs of scattering data. Black curve is experimental data, and red curve is best fit to theory. (d) predicted particle size distributions as determined from scattering data (solid line) and image data (blue area).

### Dilute Suspensions of Sphered Red Blood Cells

Finally, we performed measurements of light scattering from human red blood cells (RBCs). Red blood cell numbers, mean size, and distribution of sizes are important factors in human health. Diseases such as malaria, thalassemia minor, anemia, as well as many others, result in abnormal size distributions of red blood cells [17], [18]. Thus, having a rapid, inexpensive method of determining these parameters on a mobile device could have a wide impact on global health, especially in areas where access to flow cytometers (a gold standard for clinical particle sizing and counting) is limited. A small volume (approximately 10 microliters) of blood was taken via finger stick and diluted 1∶1000 in a cell sphering medium (3 mg of sodium dodecyl sulphate (SDS) dissolved in 10 mL of phosphate buffered saline). Due to the elastic nature of their cell membranes, red blood cells are easily isovolumetrically transformed from their characteristic biconcave shape into spheres when an appropriate sphering agent (such as SDS) is added to the cell suspension [19]. The scattering pattern of the red blood cells is shown in Figure 4 (a). A droplet of the sphered blood cell suspension was placed on a microscope slide and the cells were allowed to settle, following which 5 images were taken of different fields of view with a 10× objective, with a representative image shown in Figure 4 (b). A cut through of the scattering data is shown in black in Figure 4 (c), along with the best fit to the data from theory shown in red. The extracted particle size distribution of the red blood cells is shown as the black line in Figure 4 (d). Aggregate data from 10 independent size predictions are given in Table 2. While observed distribution values agree well with expected values for red blood cell size and distribution width for a healthy donor, to verify this result we compared it with extracted values of cell size based on the recorded microscope images. Because the microscope images are blurred by the objective's point spread function (PSF), we first performed image deconvolution using an estimate of the image PSF in ImageJ [20]. Following this we performed an automated cell count in ImageJ, which returns the calculated area of each cell. Because each of the cells are very nearly perfect spheres, the radius can be easily calculated from the area. The histogram of calculated radii for the image shown in Figure 4 (b) is shown in blue in Figure 4 (d). Fits of the histograms for the 5 images to Gaussian functions returned values reported in Table 2. We emphasize that this method of calculating the cell size is necessarily less accurate than our scattering technique, as it is limited by the accuracy of our deconvolution and subsequent image analysis. Additionally, due to the limited microscopic field of view, it measures many fewer cells than the scattering method (approximately 1,000 cells compared to more than 100,000 cells in the scattering measurements). Nevertheless, the agreement of the two calculated distributions provides an independent confirmation of the overall accuracy of the scattering technique as applied to red blood cells. We note that our use of small scattering angles and isovolumetrically sphered cells limits our sensitivity to variations in cell shape. We further note that although in this work we report only relative particle number densities, an appropriate calibration could be performed on a very well characterized sample that would enable extraction of more clinically relevant absolute number densities.

**Figure 5:**
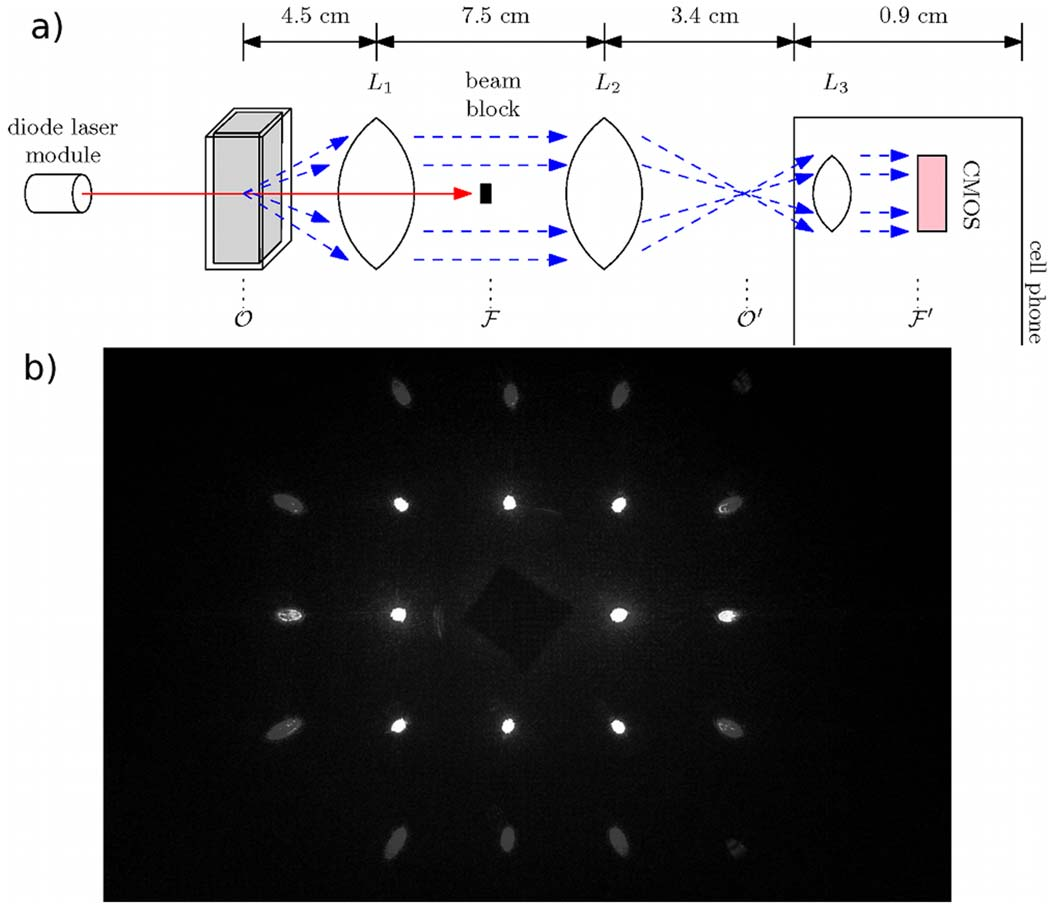
Experimental System and Calibration. (a) Schematic depiction of the experimental system. O and O' are object and image planes, respectively, while F and F' are the Fourier plane and its image, respectively. (b) Fourier image of a 200 lp/mm dual axis grating placed at O used to generate a pixel-to-angle calibration curve.

In summary, we have constructed an elastic scattering device attached to a cell phone that operates by recording angular distributions of scattered light intensity from a suspension of small particles. Experiments on a wide range of samples with sizes ranging from 0.5 to 10 microns, and with shapes ranging from nearly perfect spheres to relatively oblong prolate spheroids show the wide applicability of such a device. Extracted diameters agree better than 20 nm in the case of known size standards. We have shown results with relevance to food inspection, process control, and human health, especially those applications where inexpensive and field-portable diagnostics are of interest. Although our device may not be as powerful as flow-based particle sizers, and certainly cannot provide all of the information currently outputted by clinical hematology analyzers, it can still provide many of these parameters with the added advantages of low cost and increased portability.

## Materials and Methods

### Instrumentation

Our device, shown schematically in Figure 5 (a), consists of a 1 mW, collimated, 655 nm diode laser (AixiZ, Houston, TX) that illuminates a 4 mm pathlength plastic cuvette, with 1 mm thick walls, filled with a liquid particle suspension. Scattered and unscattered light is collected by a 45 mm focal length achromatic doublet (L1) placed one focal length from the cuvette. At the Fourier plane of the doublet we place a small (approximately 2 mm square) piece of black aluminum foil (“blackwrap”) to block the unscattered laser light. A 30 mm focal length doublet (L2) along with the 4 mm focal length internal lens of an iPhone 4 (L3, Apple, Cupertino, CA) act as a 4f imaging system, relaying the Fourier plane of L1 to the CMOS detector array of the cell phone. The sensor has a physical size of 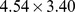 mm, with pixel dimensions of 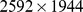 composed of 1.75 micron pixels.

Spatial locations in the Fourier plane correspond to scattering angles in the sample plane [21]. Observation of the Fourier plane, therefore, yields the distribution of scattered intensity versus azimuthal and polar angles in the sample plane. The maximum polar angle collected by the system is restricted by the limiting aperture of the cell phone camera lens. In our system the maximum angle is given by 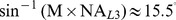, where M is the ratio of focal lengths between L1 and L2 and 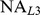 is the numerical aperture of L3. This is confirmed by placing a 200 lp/mm dual axis diffraction grating (Edmund Optics, Barrington, NJ) in the object plane of the system. The resulting Fourier image is shown in Figure 5 (b), where we can see the characteristic two axis diffraction pattern of evenly spaced points, with the second diffraction order (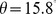) just passed by the system. Using the grating as a calibration standard, we convert the pixel positions of the image to polar angles. We note that in subsequent experiments a Snell's law correction must be made to the grating angles to account for the fact that the particles are dispersed in water and undergo refraction at both the water-cuvette and cuvette-air interface. Assuming the cuvette faces are roughly plane-parallel, the refraction can be considered to be occurring at a liquid-air interface. The correction between the detected scattering angle and true scattering angle is thus:
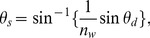
(1)where 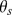 is the scattering angle of the light from the particle, 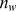 is the refractive index of the liquid used to suspend the particles, and 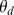 is the detected scattering angle of the light after refraction.

### Data Analysis

The camera records images of the Fourier plane of the optical system. Although the analysis performed here could be implemented on-board the mobile device, in this proof-of-concept study, images were taken from the phone and processed off-line on a desktop computer using MATLAB (The MathWorks, Natick, MA). For the nearly spherical samples of the sizes explored in this paper, the scattering data exhibits rotational symmetry. This is useful in situations in which portions of the Fourier plane are corrupted by artifacts arising from reflection from cuvette faces or other elements within the system. Taking one-dimensional radial slices of the data yield curves of intensity vs. angle that can be compared to theoretical curves generated by Mie theory. To generate these slices we took regions of the image of the size and shape shown by the green rectangle in Figure 1 (a) and averaged the values along the short dimension of the rectangle to yield the 1-dimensional curve of intensity versus angle. To obtain multiple independent predictions of particle size, and to avoid artifacts that contaminated some regions of some images (for example the left hand side of Figure 1 (b)), this average was performed for the same range of 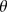 angles, but at 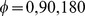 or 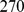 degrees. To determine the particle size distribution that best fits the data, we first created a matrix, I, of Mie scattering intensities versus polar angle and radius. The angle dimension of I spans the angles recorded by our system, truncated at 14 degrees to avoid the off-axis aberrations visible at the edge of the field in Figure 5 (b). The radius dimension explored particle sizes between 1 and 10 microns with 10 nm spacing, taking into account the known refractive index of polystyrene. We then assumed a Gaussian distribution, D, of particle sizes, and created a test function, T, that was compared to our experimentally acquired data:
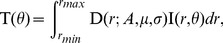
(2)where A, 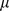, and 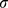 are the amplitude, mean, and standard deviation of the distribution function D. Starting from a random initial guess of the distribution parameters for D, the values of A, 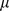, and 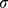 were optimized by a downhill simplex search, using the sum squared error between the test function and experimental data as the optimization metric. The result of this fitting is a predicted particle size distribution, D, whose theoretical scattering pattern best fits the data. We note here that error surfaces in inverse scattering problems can be highly degenerate in the limit of many free parameters, or (like in the milk experiments shown above) when the scattering data itself is fairly featureless and patterns are distinguished merely by slopes and intercepts. Therefore, if some prior knowledge of the sample is at hand, it is worthwhile to include it in the fitting process. For the data shown in this paper, expanding the search space to include larger and smaller particle sizes did not change the fits, however we do make an assumption about our sample in using a single Gaussian or log-normal distribution to model the scatterers. We have also tried fitting the data with model-free techniques, regularizing the fit with total variation and sparsity constraints (enforcing smooth functions that have many zeros). These produce acceptable number density curves (data not shown), but lack the accuracy we see with our current model-based fits.
